# Factors associated with the closure of German obstetric units and its effects on accessibility

**DOI:** 10.1093/eurpub/ckac131.424

**Published:** 2022-10-25

**Authors:** J Hoffmann, N Scholten, T Dresbach

**Affiliations:** 1 Institute for Medical Sociology, Health Services Research, and Rehabilitation Science, University of Cologne, Köln, Germany; 2 Department of Neonatology and Pediatric Intensive Care Medicine, University Hospital Bonn, Bonn, Germany

## Abstract

**Background:**

An increase in regionalization of obstetric services is being observed worldwide. This study investigated factors associated with the closure of obstetric units in hospitals in Germany and aimed to examine the effect of obstetric unit closure on accessibility of obstetric care.

**Methods:**

Secondary data of all German hospital sites with an obstetrics department were analyzed for 2014 and 2019. Multivariate logistic regression was performed to identify factors associated with obstetrics department closure. Subsequently, the driving times to a hospital site with an obstetrics department were mapped, and different scenarios resulting from further regionalization were modelled.

**Results:**

Of 747 hospital sites with an obstetrics department in 2014, 85 obstetrics departments closed down by 2019. Only the annual number of live births was observed to be a factor significantly associated with the closure of obstetrics departments (odds ratio: 0·995; confidence interval: 0.993-0.997). Areas in which driving times to the next hospital site with an obstetrics department exceeded the 30 and 40 min threshold increased from 2014 to 2019. Scenarios in which only hospital sites with a pediatrics department or hospital sites with an annual birth volume of ≥ 600 were considered resulted in large areas in which the driving times would exceed the 30 and 40 min threshold.

**Conclusions:**

The annual number of live births is a factor significantly associated with the closure of obstetrics departments. Despite the closure, good accessibility is maintained for most areas in Germany. Although further regionalization may ensure high-quality care and efficiency, it will have an impact on accessibility.

**Key messages:**

• Despite the closure of many obstetric departments, regional accessibility to obstetric care remains good for most areas in Germany.

• Further regionalization may ensure high-quality care and efficiency but will have an impact on accessibility.

